# Developing Standards for Cultural Competency Training for Health Care Providers to Care for Lesbian, Gay, Bisexual, Transgender, Queer, Intersex, and Asexual Persons: Consensus Recommendations from a National Panel

**DOI:** 10.1089/lgbt.2021.0464

**Published:** 2022-07-02

**Authors:** Mandi L. Pratt-Chapman, Kristen Eckstrand, Amorie Robinson, Lauren B. Beach, Charles Kamen, Alex S. Keuroghlian, Scott Cook, Asa Radix, Markus P. Bidell, Daniel Bruner, Liz Margolies

**Affiliations:** ^1^ Department of Medicine, GW Cancer Center, The George Washington University, Washington, District of Columbia, USA.; ^2^ Department of Psychiatry, University of Pittsburgh, Pittsburgh, Pennsylvania, USA.; ^3^ The Ruth Ellis Center, Highland Park, Michigan, USA.; ^4^ Department of Medical Social Sciences, Northwestern University Feinberg School of Medicine, Chicago, Illinois, USA.; ^5^ Department of Surgery, University of Rochester Medical Center, Rochester, New York, USA.; ^6^ National LGBTQIA+ Health Education Center, The Fenway Institute, Boston, Massachusetts, USA.; ^7^ Department of Medicine, University of Chicago, Chicago, Illinois, USA.; ^8^ Callen-Lorde Community Health Center, New York, New York, USA.; ^9^ Department of Psychiatry, Hunter College, CUNY, New York, New York, USA.; ^10^ Whitman-Walker Health, Whitman-Walker Institute, Washington, District of Columbia, USA.; ^11^ National LGBT Cancer Network, New York, New York, USA.

**Keywords:** consensus-based recommendations, cultural competency standards, health care and social service, LGBTQIA+, training and education

## Abstract

**Purpose:**

Lesbian, gay, bisexual, transgender, queer, intersex, and/or asexual and other sexual and gender diverse persons (LGBTQIA+ or SGD persons) experience barriers to equitable health care. The purpose of this article is to describe a collaborative process that resulted in core cultural competency recommendations addressing training for those who provide health care and/or social services to LGBTQIA+ patients.

**Methods:**

In 2018 and 2019, Whitman-Walker Health, a Federally Qualified Community Health Center in Washington, DC, and the National LGBT Cancer Network purposively selected leaders of community clinics and community-based organizations, cultural competency trainers, and clinicians and researchers with expertise in SGD health with diverse lived experiences to develop consensus-based cultural competency recommendations. Recommendations were developed through a synthesis of peer-reviewed studies, publicly accessible curricula, and evaluations of SGD cultural competency trainings; two in-person convenings; and iterative feedback from diverse stakeholders.

**Results:**

Five anchoring recommendations emerged: (1) know your audience; (2) develop and fine-tune the curriculum; (3) employ both adult and transformational learning theories; (4) choose multiple effective trainers; and (5) evaluate impact of training. These recommendations promote an ongoing process of individual and organizational improvement and a stance of humility rather than competence to be mastered.

**Conclusion:**

By setting core cultural competency standards for all persons involved in health care and social services, these recommendations complement existing clinical competency recommendations to advance SGD health equity.

## Introduction

In the past 15 years, awareness has broadened regarding the importance of tailored health care for individuals minoritized based on sexual orientation, sexual attraction, gender identity, gender expression, and differences in sex development.^1,2^ Individuals who are lesbian, gay, bisexual, transgender, queer, intersex, and/or asexual (LGBTQIA+) and other sexual and gender diverse (SGD) persons of all races and ethnicities experience barriers in accessing and receiving equitable health care. Many SGD people are multiply minoritized based on race, ethnicity, economic status, and other social factors.^3^ The impact of multiplied stigma can be exponential. Due to these intersectional disparities, more jurisdictions and schools require health professionals to undergo SGD cultural competency training^4,5^; however, no national standard for such training currently exists.

Cultural competence in health care initially aimed to address health disparities^6–8^; yet early models were theoretically flawed.^9^ First, models suggesting that a provider can have “mastery” over another person's lived experiences is patriarchal, racist, and classist.^10,11^ Second, competence focused at the individual level does not adequately address the interdisciplinary way that care is delivered.^12^ Cultural competence must include all levels of employees within institutions and move beyond individual competence to organizational culture, protocols, and policies. Third, models of competence have prioritized the role of providers over other employees; however, barriers to accessing care encompass factors that precede clinical interactions (e.g., discrimination in waiting rooms),^13^ suggesting a need for competence across all roles in health care. Finally, solely focusing on common processes impacting health equity across diverse groups (e.g., economic strain) is insufficient.^14^

Because different identity groups experience challenges to health equity in unique ways, affirming care must be adaptable.^15^ This is particularly important for understanding how health inequity might influence those who are minoritized based on intersectional demographics or lived experiences.

The concept of cultural competence has evolved because of these critiques, with a specific focus on *process* over *outcome*. Cultural and narrative humility are processes that are grounded in the acknowledgement that each patient's story is different and requires health care provider self-reflection, self-evaluation, and self-critique.^11,16^ While cultural humility is often discussed with cultural competence, policy and legislative mandates often use the term “cultural competence.”^4^

The difference between assumed competence and humility is critical and highlights the importance of trainers who must balance *outcome* mandates with the use of *processes* known to be effective. We use the term “cultural competence” to align with mandates, given that they are the vehicle that requires SGD cultural competency training; however, we endorse a stance of cultural humility. Indeed, we support the use of the term cultural humility and encourage mandates to embrace this more appropriate term in the future.

Numerous national organizations have created standards for culturally responsive health care. Since 2008, the Human Rights Campaign's Healthcare Equality Index has assessed hospitals' provision of services for SGD patients and families and support for SGD employees.^17^ The Joint Commission released recommendations for health care organizations to create an inclusive climate for SGD patients.^18^

The Association of American Medical Colleges defined clinical competency recommendations for medical doctors,^2^ with position statements from the American Medical Association (AMA)^19^ and American Nurses Association (ANA)^20^ following suit. The American Psychological Association (APA) has a long history of developing SGD-affirming policies, and the APA Task Force on Psychological Practice with Sexual Minority Persons published SGD guidelines, approved by the APA Council of Representatives.^21^ The authors also examined the role of gender identity and expression as a key aspect of intersectionality. Furthermore, the American Counseling Association's Society for Sexual, Affectional, Intersex, and Gender Expansive Identities published LGBQIA^22^ and transgender^23^ competencies.

SGD clinical competency recommendations are appropriately related to the clinical scope of the discipline for which the recommendations are made. Yet given that nonclinical employees encompass over 30% of health care jobs,^24^ and that people access social services outside the health care setting, it is essential that all health care and social service staff receive high-quality cultural competency training to effectively address basic SGD health needs.

This article describes a national collaborative process that resulted in a set of core cultural competency recommendations to inform training practices that focus on health care services to SGD patients.^25^ The goal of these recommendations is to increase knowledge, skills, and positive attitudes of health care personnel when providing services to SGD patients, partners, and their families. This article describes the methods used to develop these standards to ensure transparency and credibility for those considering use of the recommendations.

We acknowledge that people with intersex conditions and asexual persons do not always identify as part of the LGBTQ+ community; however, we have included these communities to raise awareness of shared experiences of stigma and barriers to health care. We focused specific attention on including perspectives of those with intersex conditions and persons with asexual lived experience; yet engagement of persons within both of these communities could have been greater. We use the term SGD to be as inclusive as possible, acknowledging that many people do not identify with the terms represented in the LGBTQIA+ abbreviation.

Our aim is to be inclusive and highlight the role of power in stigmatizing persons minoritized in multiple and overlapping ways. While we know our recommendations will need refinement, they are built on processes that require humility in understanding various lived experiences and engaging community members to improve the health of SGD persons in their own community.

## Methods

### Participant selection

In 2018 and 2019, Whitman-Walker Health and the National LGBT Cancer Network purposively selected leaders of community clinics and community-based organizations, cultural competency trainers, and clinicians and researchers with expertise in SGD health from across the United States from both rural and urban settings and with lived experience to develop consensus-based SGD cultural competency recommendations. Per Whitman-Walker Health policies, no institutional review board approval was sought for this project as it was considered quality improvement.

Participants were selected based on lived experience as an SGD person, with intentionality of including SGD people of color, as well as professional leadership in developing cultural competency curricula, education, and trainings. We attempted to optimize participant diversity in professional role, race/ethnicity, gender identity, and sexual orientation. Of the summit participants (*N* = 30), 6 uniquely participated in the first summit, 8 uniquely in the second summit, and 16 attended both summits.

Twenty-two participants completed a demographic questionnaire to help us describe the diversity of people engaged (Table 1). To push back against erasure of identity that occurs when forcing individuals to select mutually exclusive demographic categories and in the spirit of intersectionality, Table 1 summarizes demographics based on participants' own nonmutually exclusive descriptions of their overlapping lived experiences.

**Table 1.** Summit Participant Characteristics (*n* = 22)

Participant characteristic	*N* (%)
Professional role (select all that apply)	
Educator	6 (27.3)
Health care provider	6 (27.3)
Patient Advocate	5 (22.7)
Researcher	5 (22.7)
Professional setting	
Academic	12 (54.5)
Community based	10 (45.5)
Gender (select all that apply)	
Man	6 (27.3)
Woman	10 (45.5)
Cisgender	8 (36.4)
Transgender	2 (9.1)
Genderqueer	1 (4.5)
Nonbinary	3 (13.6)
Other: Demigender	1 (4.5)
Sexual orientation (select all that apply)	
Asexual	1 (4.5)
Bisexual	2 (9.1)
Gay	8 (36.4)
Lesbian	7 (31.8)
Queer	11 (50.0)
Questioning	1 (4.5)
Same-gender loving	2 (4.5)
Straight/Heterosexual	2 (9.1)
Race/ethnicity (select all that apply)	
American Indian/Alaska Native	2 (9.1)
Asian	2 (9.1)
Black/African American	5 (22.7)
Hispanic/Latino	2 (9.1)
Middle Eastern or North African	1 (4.5)
White	13 (59.1)

May not total 100% due to categories not being mutually exclusive.

All summit participants were offered the option of working toward developing an article on summit recommendations with transparency that contribution would lead to authorship. Article authors were those who opted in; all others opted for acknowledgment.

### Guiding theoretical frameworks

Multiple theoretical frameworks guided the *content* and the *process* of these recommendations. Recommendations were based on five principles: (1) health care is interdisciplinary and cultural competence recommendations must address the range of personnel engaged in health care systems; (2) there is a “core” to SGD cultural competence; (3) cultural humility is fundamental and a lifelong goal—not an achievement at a point in time; (4) community engagement is critical; and (5) qualified, diverse trainers are crucial.

Development of these recommendations was guided by *intersectionality* and *minority stress theory*.^26^ Intersectionality examines the ways in which individuals are impacted by intersecting processes of power imbalances that influence inequity either positively or negatively.^14^ Minority stress theory specifically explores distal (i.e., external to oneself, such as harassment and discrimination) and proximal (i.e., internal to oneself, such as internalized shame) forces related to one's identity and/or minoritized status that create stress for an individual.^27,28^

Regarding implementation of recommendations, adult and transformational learning theories are suggested pedagogic strategies. Adult learning theories describe processes and conditions by which adults effectively engage in lifelong learning.^29^ Transformational learning theories seek to understand the processes by which learners interpret new information in the context of past ideas and understandings to shift their worldview through critical reflection.^30^ As we recognize the diversity of health care employees' professions, ages, and experience, adult and transformational learning theories are paramount to considering how to support all employees in building SGD cultural competence. Such diversity also supports selection of multiple trainers who represent intersectional experiences of SGD persons.

### Process of developing recommendations

Recommendations were developed through four phases. Participants (*n* = 22 in 2018; *n* = 24 in 2019; *N* = 30) convened on two occasions, in person, for a 2-day summit (October 5–6, 2018 and November 8–9, 2019). At the first summit, participants reviewed a synthesis of peer-reviewed studies, publicly accessible curricula, and evaluations of trainings, while sharing their own experiences. Participants recommended best and promising practices. Then feedback was gathered from diverse additional stakeholders (*n* = 63). Recommendations were refined at the second summit, which included additional participants who represented greater racial and ethnic diversity than those in the first summit, and highlighted lived experiences of intersex and asexual persons.

## Results

Implementation strategies for each of five major recommendations resulted (Table 2). The major recommendations are consistent with, and reiterate, known best practice recommendations for developing a training, providing face validity.^31^ The strategies for each recommendation highlight the unique and necessary components for SGD cultural competency training.

**Table 2.** Sexual and Gender Diverse Cultural Competency Recommendations and Implementation Strategies

Recommendation	Implementation strategies
(1) Prepare for a training: Know your audience	Conduct an SGD-focused needs assessment to determine goals of training (e.g., assess current knowledge, skills, abilities, policies, procedures, and culture). Who are the champions of the SGD training? Why are they prioritizing training now? What is the degree of leadership support for actions to improve organization-level cultural competency?
(2) Develop and fine-tune the training curriculum	Provide foundational information on SGD concepts, terminology, culture, discrimination, and health disparities; health promotion strategies; and intersectionality. Facilitate learner self-awareness of assumptions and biases. Teach communication skills to optimize respectful shared decision-making. Avoid stereotypes and generalizations, encourage resiliency. Describe local and federal laws affecting SGD persons' social determinants of health and health care. Include organizational environment, policies, and processes that are welcoming and unwelcoming to SGD patients. Name what is not being covered.
(3) Employ the most effective methods of delivery: Adult learning and transformational learning	Encourage learners to identify their own learning needs. Ask learners to share expertise related to SGD cultural competence. Facilitate understanding of learners' culture, values, and history related to SGD communities. Encourage learners to disrupt old patterns of meaning and create new understanding. Motivate behavior change based on new understanding. Use multiple modes of interactive learning (multimedia, case studies, narrative, and self-reflection). When possible, provide follow-up sessions to reinforce content and skills development.
(4) Choose the right trainers and use them effectively	Coordinate training among multiple trainers who represent diverse lived experiences. Choose trainers with expertise on SGD health and health care, lived experience, skill addressing implicit and explicit bias, and ability to respond to strong emotional reactions. Acknowledge and state the limitations of trainers to meet the expected needs. Compensate trainers fairly, especially nonacademic community member trainers.
(5) Evaluate the training	Options: number of learners, demographics, satisfaction with content and trainers; knowledge, attitudes, skills change; intention and motivation to change, actual behavior change, organizational change.

SGD, sexual and gender diverse.

First, SGD cultural competency training should be designed with consideration for a specific audience of learners. Organizations should clarify how training for SGD cultural competence became a priority, what the motivation is for leadership and trainees to attend, the current state of knowledge among their potential learners, the current organizational culture, and a plan to reinforce training through follow-up activities.

Second, organizations and trainers should develop and refine content based on the learner and organizational assessment. Setting goals for knowledge, attitudes, and behavioral change helps focus the curriculum. Time constraints are also a factor. Trainers should name what is included in a training, what is not, and why. Extensive details on potential topics and curricula are provided in the publicly accessible online guide.^25^

Third, adult learning and transformational learning approaches can optimize and reinforce learning. Adult learning theory relies on learner reflection to identify gaps in knowledge and learning goals, as well as experiential learning. Prioritizing goals for the training with learners can set expectations and give learners some control over their experience. Use of examples likely to be encountered by learners in their specific clinical settings will reinforce training relevance. It is important to acknowledge that SGD trainings are likely to result in some strong reactions as learners are encouraged to examine assumptions and implicit biases (i.e., unconscious thoughts, feelings, and attributions toward a type of person or group of people) on which they have not previously reflected.

Transformational learning theory leans into uncomfortable emotional responses to challenges and critically explores habituated thinking patterns. Trainers should verbally acknowledge to trainees the role of discomfort during the learning process, help prepare them for potential reactions, and invite them to embrace their curiosity around these reactions.

Fourth, organizations should be thoughtful about the fit of trainers to organizational and employee needs. Multiple trainers with maximum variation of lived experiences can inform the broader diversity of SGD lived experiences. Choosing trainers with experiences that might mirror patients seen within the organization can optimize relevance of lessons learned. Acknowledging limitations of trainers—both in terms of experiences and expertise—is important. In addition, coordinating with multiple trainers can provide important respite for trainers when emotions are distressing and curricula are challenging to facilitate. Inclusion of community voices that are not academic or professional is especially encouraged and important.

Inclusion of local community-based organizations that serve SGD clients can create and strengthen long-term relationships to reinforce affirming care for SGD patients for a particular organization. In addition, local organizations are likely to be attuned to the nuances of SGD persons' needs in the context of their specific geography, political landscape, and local mandates. Providing fair compensation of trainers and community members participating in training is important to convey the value of their expertise and appreciation for the time and emotional labor of sharing knowledge that could benefit the organization and improve learners' professional behaviors.

Fifth, evaluate the training. There are few extant evaluations of SGD cultural competency training.^32,33^ Evaluating training sessions can help organizations model the regular collection of data for quality improvement to support their commitment to providing SGD-affirming care and striving for culturally competent care. Understanding the impact of training on learner knowledge, attitudes, skills, intention to change, motivation to change, and actual behavior change can refine ongoing professional development approaches and add to the evidence base in the literature. The *Transforming Healthcare: A Guide to Best Practices in LGBTQIA+ Cultural Competency Training*^25^ offers detailed support for the high-level recommendations presented in Table 2, as well as examples of training curricula, publications, research, documentaries, toolkits, worksheets, workshop exercises, surveys, and websites. In addition, the interactive website includes a glossary of key terms and details on the origins and the development of recommendations, including names and affiliations of summit participants and reviewers.

For those interested in rigorous evaluation, two examples of SGD-specific validated scales that could be used to evaluate trainings include the Lesbian, Gay, Bisexual, Transgender Development of Clinical Skills Scale (LGBT-DOCSS)^34^ and the QUeering Individual and Relational Knowledge Scale (QUIRKS).^35^ Other methods of evaluation could include role play with feedback, use of standardized patient scenarios, and nonvalidated measures that ask learners to rate their confidence in performing learning outcomes before and after training and/or motivation to change behaviors.^33^

## Discussion

Although a growing number of educational programs and health systems offer SGD training, there is a dearth of standards for such training. *Transforming Healthcare: A Guide to Best Practices in LGBTQIA+ Cultural Competency Training*^25^ intends to fill this gap. This guide provides recommendations from experienced trainers, employees, providers, and community members. The core of high-quality SGD cultural competence includes the following: using current knowledge, skills, and attitudes of learners; reasons for training being prioritized; the organizational environment; and the amount of time available**.** The guide does not mandate specific content to be covered in training, but rather provides a guide to optimize SGD cultural competency training decisions from planning through implementation and evaluation.

While we believe these recommendations to be useful to any who are involved in the process of developing, disseminating, and evaluating training, they are particularly important for those leading training from start to finish. The guide^25^ and this overview of its development suggest processes and recommendations to maximize impact of SGD-specific training. We recommend instructors use these recommendations when designing their trainings. Leaders can consider processes before, during, and after training to ensure maximum benefit.

These recommendations are complementary to existing discipline-specific and organizational standards.^17–20,36,37^ In addition to setting a standard for core LGBTQIA+ cultural competence, they are intended to aid organizations in selecting trainers and to assist trainers in encouraging learner reflection, stereotype negation, and depathologizing minoritized identities. Organizations should reference complementary clinical competency work, position statements from discipline-specific membership organizations, and organizational baseline standards when using these recommendations (Table 3).^2,17,18,20,21,36,37^

**Table 3.** Guideline Matrix

Form of competence	Recommending organization				
	AAMC^2^	APA^21,36,37^	ANA^20^	Joint Commission^18^	HRC HEI^17^
Organizational				✓	✓
Individual (cultural and clinical)					
Physician	✓				
Nurse			✓		
Mental Health		✓			

AAMC, Association of American Medical Colleges; ANA, American Nurses Association; APA, American Psychological Association; HRC HEI, Human Rights Campaign Healthcare Equality Index.

Specifically, the current recommendations form a core of cultural competence from which existing discipline-specific and organizational standards can build. Importantly, the current recommendations for SGD *cultural* competence are distinct from recommendations for *clinical* competence that delineate the clinical care needs for SGD populations.

Importantly, these recommendations are distinct in several ways. First, the current recommendations recognize the importance of and set criteria for how to select a trainer for SGD cultural competency training. These include recommendations for using multiple trainers with diverse identities and lived experiences, and compensating trainers fairly. Second, these recommendations set a standard for what *all* employees in health care systems and social service organizations should be competent in with respect to SGD populations. Setting uniform standards is critical for shared accountability, confidence in co-workers, and establishing what is unacceptable in health care spaces. The latter allows for any individual in the health care system to be able to recognize inappropriate behaviors and intervene accordingly. Finally, our recommendations set core competency standards that include the processes inherent to cultural humility. This approach allows a framework for improving the competence of health care systems to better serve SGD colleagues and patients.

### Strengths

A particular strength of these recommendations is the commitment to nonclinical SGD community engagement. These recommendations include critical feedback from nonclinical members across SGD communities. Recently, numerous calls have been made for more community engagement in clinical education.^38^ To our knowledge, the present standards are the first to embody this commitment for SGD communities.

### Limitations

We are acutely aware of the historic, social, and political context for this work. These recommendations cannot adequately address the experiences of every SGD person. They offer an approach to training, which includes learning about unique experiences of SGD people. Attempts were made to be inclusive of SGD subpopulations; however, we were limited by those who were publicly visible and willing and able to provide feedback. The recommendations require strengthening in perspectives from asexual individuals; those who are intersex; First Nations/Two Spirit people; and those from other cultural backgrounds. We support and acknowledge that ongoing and iterative refinements are necessary to reduce disparities faced by SGD people.

Skills building takes multiple sessions. These recommendations are not meant to produce rapid change; however, they can help guide individuals and organizations toward meaningful and sustainable change. Understanding equity, and compassionately and effectively serving the needs of SGD persons and families, requires an ongoing commitment not only by providers but also by health care systems. System-level interventions are necessary to truly create an inclusive health care environment. It is helpful for organizations to develop a long-term relationship with trainers that encompasses a series of training sessions over a period of time and identify staff champions to optimize advancements in implementing and improving SGD-affirming organizational policies and practices.

## Conclusion

*Transforming Healthcare: A Guide to Best Practices in LGBTQIA+ Cultural Competency Training*^25^ complements existing guidelines on SGD cultural competency training by highlighting core components and their application to all individuals within health systems. Alongside clinical and organizational SGD competency frameworks, these recommendations can strengthen delivery of health care and, ultimately, improve health care experiences and outcomes for SGD patients.
